# Understanding linkage to care with HIV self-test approach in Lusaka, Zambia - A mixed method approach

**DOI:** 10.1371/journal.pone.0187998

**Published:** 2017-11-17

**Authors:** Jenala Chipungu, Samuel Bosomprah, Arianna Zanolini, Harsha Thimurthy, Roma Chilengi, Anjali Sharma, Charles B. Holmes

**Affiliations:** 1 Centre for Infectious Disease Research in Zambia (CIDRZ), Lusaka, Zambia; 2 Department of Biostatistics, School of Public Health, University of Ghana, Accra, Ghana; 3 American Institutes for Research, Lusaka, Zambia; 4 Gilling’s School of Public Health, University of North Carolina at Chapel Hill, North Carolina, Chapel Hill, United States of America; 5 School of Medicine, Johns Hopkins University, Baltimore, United States of America; New York University, UNITED STATES

## Abstract

**Introduction:**

HIV self-testing (HIVST) is a novel approach designed to assist in achieving the goal of at least 90% of the population that learn their HIV status. A self-test user with a positive test is required to visit a clinic to link into HIV care, yet little is known about patient preferences for linkage strategies. We examined the intention to link to care amongst potential HIVST users and the suitability of three linkage to care strategies in Lusaka Province, Zambia.

**Methods:**

We conducted a representative cross sectional survey of 1,617 individuals aged 16–49 years old in Lusaka Province. Participants were shown a video of the HIVST. Data on intention to link to care and preferred linkage to care strategies—text message, phone call and home visits were collected. Eight focus group discussions were held concurrently with survey respondents to understand their preferences between the three linkage to care strategies.

**Results:**

Of 1617 enrolled, 60% were women, 40% were men, with an average age of 27years (IQR = 22, 35). More men than women had at least secondary education (84% vs 77%) and were either employed or self-employed (67% vs. 41%). 85% (95%CI = 83 to 86) of participants said they would link to care within the first week of a positive self-test. Income >2,000 Kwacha (USD 200) per month versus income < 2,000 Kwacha (Adjusted odds ratio (AOR) = 0.59; 95%CI: 0.40 to 0.88; p = 0.009) and never versus prior HIV testers (AOR = 0.54; 95%CI: 0.32 to 0.91; p = 0.020) were associated with reduced odds of intention to link to care. 53% (95%CI = 50 to 55) preferred being prompted to link to care by home visits compared to phone call (30%) or SMS (17%).

**Conclusion:**

We found almost nine out of ten potential HIVST users in the general population intend to link to care shortly after a positive test, and preferred home visits or phone calls to facilitate linkage, rather than SMS. Also, higher income earners and those who never tested for HIV were associated with reduced odds of intention to link to care. Policy guidelines and implementation strategies for HIVST should be responsive to patient preferences for linkage to care strategies to achieve the continuum of HIV care.

## Introduction

Globally, a significant number of people do not know their HIV status [1]. UNAIDS has set an ambitious 90-90-90 target where 90% of people living with HIV (PLHIV) know their status; of these, 90% are linked to antiretroviral therapy (ART), and 90% of those on ART achieve viral suppression [2]. Close to 70% of men and women did not know their status prior to population-based surveys in select countries in sub-Saharan Africa [1]. In Zambia, the 2014 Demographic and Health Survey (DHS) revealed that only 37% of men and 46% of women knew their HIV status despite over a decade of HIV testing and counselling (HTC) implementation in the country [3]. New strategies and approaches are needed to raise the proportion of men and women testing to 90% in Zambia, particularly among those marginalized in society.

HIV self-testing (HIVST) is a novel approach that has potential to contribute to bridging the gap between the UNAIDS target and the current HIV testing rates across the world [4, 5]. HIVST allows persons to collect their own blood or saliva sample, test it and interpret their own result [6]. Studies have shown high acceptability rates for HIVST in the general population across different settings including low middle-income countries (LMICs) such as Kenya and Malawi [7, 8]. A qualitative study among key populations in Cambodia reported high willingness to use HIVST and acceptability amongst transgender women, men who have sex with men, and female entertainment workers [9]. High acceptability of HIVST was reportedly due to convenience, privacy, prompt test result and confidentiality [8–11]. However, there were concerns about the ability of lay people to perform the test in LMICs. A multi country study conducted in Malawi, Kenya and South Africa found that after being given written and pictographical instruction sheets in English and local language, less than 25% of study participants performed each step of the self-test correctly [12].

Linkage to HIV care after taking a self-test is a further issue of serious concern, given that the tests may be performed in the community without supervision. Although HIVST models have the potential to increase the proportion of people knowing their status, there is concern that they may exacerbate already poor rates of linkage to care (15). Given the absence of self-testing in Zambia to date, it was unknown how patients would respond to self-testing, and what linkage strategies they would prefer. We therefore undertook a large representative survey along with additional qualitative work, in order to examine intention to link to care among potential HIV self-testers, and to explore the suitability of several possible prompts to link to care.

## Methods

### Ethical considerations

The study research protocol was jointly approved by Excellence in Research Ethics and Science (ERES) Converge in Lusaka, Zambia and the University of North Carolina, Chapel Hill, USA Institutional Review Boards. All participants were informed of the risks and benefits of participating in the study and written consent was obtained before being interviewed. Written assent was obtained from participants aged 18years and below and additional written consent was obtained from their guardians. Where participants were illiterate, literate witnesses were present during the consenting process to confirm that the information being given was accurate and thumbprints were obtained from participants.

### Study setting

The study was conducted in Lusaka, Chongwe and Kafue Districts of Lusaka Province, Zambia. The province is the most densely populated with a population of 2.1million and an HIV prevalence rate of 16.3% [3].

### Study design

This study used a convergent design, including concurrent implementation of qualitative and quantitative methods and merging the data to interpret study findings [13]. The quantitative data was collected through a cross sectional survey whereas the qualitative data was collected through focus group discussions (FGDs).

### Sample size consideration

We assumed that 40% of adult residents in Lusaka would link to care within one week if HIVST result was positive. Given that 45% of the Zambian population was adults aged 16–49 years, we therefore required a total of approximately 1,546 households to estimate prevalence of linkage to care with 0.1 precisions, adjusting for 1.15 design effect and a nonresponse rate of 0.8%. A household size of one was incorporated because only one individual per household was included in the study. The Demographic and Health Survey (DHS) program sample size formula was used for this estimation.

With the intention of obtaining variability and saturation, a total of eight FGDs across urban and rural areas were planned. Each FGD had eight participants aged 16–49 years. Females were recruited into four FGDs separate from males to avoid power relations stemming from cultural expectations.

### Sample design and sampling procedure

The sample for this study was a stratified sample selected in two stages using the 2010 population and housing census as the sampling frame. Because Lusaka District is much more urban than the other districts within Lusaka Province and contains the majority of individuals in Lusaka Province, the census supervisory areas (CSAs) were stratified into ‘Lusaka district’ and ‘non-Lusaka district’. To ensure that the entire province is more uniformly represented in the survey, 13 CSAs were allocated to Lusaka District and 4 CSAs to non-Lusaka districts. Samples were selected independently in each stratum, using a two-stage selection process. In the first stage, the 17 CSA were selected with probability proportion to the CSA size. The CSA size is the number of households residing in the CSA.

In the second stage of selection, a fixed number of 100 households per CSA were selected systematically using linear or spiral approaches. Maps of the CSAs were subdivided into smaller areas and assigned numbers, which the research team used to randomly select a starting point and traced houses based on the geographical setting and location of houses, and approached every 3^rd^ house up to approximately 100 households in each of the 17 CSAs. The survey interviewer was instructed to interview only one eligible participant per household. An individual was eligible to participate in the study if (s)he was 16–49 years of age. At each household, all eligible participants were assigned a serial number and selected using a computer-generated list of random numbers. In the event that the selected person was not at home, a maximum of 3 repeat visits was attempted to obtain complete data. If unsuccessful after three attempts, the household was marked as ‘non-response’ and there was no replacement by another eligible person in the household. Our non-replacement strategy was to ensure that the sample remain random and representative of the target population.

Convenience sampling was used to select participants for FGDs. Every fourth participant surveyed was asked to participate in FGDs. If consented, they collected their phone numbers and communicated a central location, a day and time for the FGD. If consent was denied the next survey participant was asked.

### Data collection procedures

A locally produced video of an oral fluid-based HIV test [14] was used to introduce the HIVST and to standardize the information provided to participants and limit interviewer-specific discrepancies. To assess participant’s intention to link to care, they were asked to imagine that “After a self-test you are supposed to go to the facility. If you did a self-test and it was positive, when would you go to the health facility?”. In answering this question they were encouraged to choose the following alternatives: a) within that same week b) after the first week but before feeling sick or c) until feeling sick. To assess linkage to care strategy, the participants were asked “What would make you more likely to go the health facility for confirmatory testing?” and were encouraged to choose from the following alternatives: a) received a test message (SMS), b) received a phone call, or c) received a home visit from a health care worker.

The FGD guides (S1 FGD Guide) were used to understand the reasons why participants preferred the use of either text message, phone call or home visit as a linkage to care strategy. Vignettes were used to depict the process of follow-up via the three linkage to care (follow up) strategies. Participants were asked which follow-up strategy they preferred and why.

All instruments were administered in the preferred languages of the respondents and survey data were collected on tablets. FGDs were voice recorded and later transcribed. We conducted 8 FGDs with females and males aged 16–49 years. Amongst the 8, 4 were conducted in the urban areas and the other 4 in rural areas. FGDs with females were held separately from males to avoid power relations. Each FGDs was attended by 8 people, therefore there were a total of 64 FGD attendees.

### Data analysis

The primary outcome was intention to link to care defined as the fraction of people who reported being willing to link to care within a week of self-testing positive. Key background characteristics were stratified by sex. Chi-square test was used to assess possible association of each of background characteristics with linkage to care. Continuous variables were categorized to understand how the outcome was distributed across sub-populations. Multivariable logistic regression model was used to identify variables that are independently associated with odds of intention to link to care. Variables were removed at p-value greater than 0.2 using backward stepwise selection. Likelihood ratio test was used to calculate p-value. In a secondary analysis we explored association of background characteristics with follow up strategy, defined as the fraction of respondent who preferred a home visit from a health care worker as a follow up method. All analyses were adjusted for the survey design characteristics (sampling weight and stratification). Since only one individual was included per household, there was no adjustment for clustering within a household. All analyses were performed using Stata 14 MP (StataCorp, College Station, Texas, USA).

Two coders listened to voice recordings and read printed copies of transcripts in order to become familiar with the data. Soft copy transcripts were imported into NVIVO 10 and analysed using thematic content analysis. Identifying information on the transcripts were removed prior to analysis. Coding was done under three emerging themes, namely text message, phone call and home visits. Findings were then categorized under ‘reasons for linkage to care strategy’ and ‘reasons against linkage to care strategy’.

## Results

1,617 respondents participated in the survey (Table 1). Of these, 970 (60%) were women and 647 (40%) were men. The median age for all participants was 27 years (IQR = 22, 35). More men than women had at least secondary education (84% vs 77%) and were either employed or self-employed (67% vs. 41%; Table 1). About 77% of men reported having had an HIV test 12 months prior to the survey compared to 90% of women. Acceptability of HIVST was similar between men and women (Table 1). 87% of women and 83% of men reported being willing to link to care within one week if an HIVST result was positive compared to 87% of women, whereas and 55% of women and 51% of men preferred a home visit linkage strategy. compared to 55% of women (Table 1).

**Table 1:** Characteristics of and reported linkage to care, and follow up strategy among respondents aged 16–49 years in Lusaka province, Zambia.

Characteristics	Women (%)	Men (%)	Total (%)
	N = 970	N = 647	N = 1617
**Age (Years)**			
16–24	369(38)	202(31)	571(35)
25–34	369(38)	257(40)	626(39)
35–49	232(24)	188(29)	420(26)
**Education N (%)**			
No Education	33(3)	14(2)	47(3)
Primary Education	196(20)	88(14)	284(18)
Secondary Education	647(67)	409(63)	1056(65)
Tertiary Education	94(10)	135(21)	229(14)
**Monthly income (Kwacha)**^**1**^			
<500	111(29)	64(15)	175(11)
501–1000	127(33)	100(24)	227(14)
1001–2000	67(17)	103(24)	170(11)
2001–5000	60(16)	121(29)	181(11)
>5000	19(5)	33(8)	52(3)
**Employment status**			
Employed	130(13)	241(37)	371(23)
Self-employed	270(28)	193(30)	463(29)
Unemployed	570(59)	213(33)	783(48)
**HIV Tested**			
Yes	875(90)	500(77)	1375(85)
No	95(10)	147(23)	242(15)
**HIVST Acceptability**			
Very Comfortable	725(75)	473(74)	1198(74)
Somewhat Comfortable	157(16)	106(17)	263(16)
Not Comfortable	83(9)	61(9)	144(9)
**Linkage to care**			
Within a week	838(87)	533(83)	1371(85)
After a week	120(12)	98(15)	218(13)
Until illness	12(1)	16(2)	28(2)
**Follow up strategy**			
SMS	157(16)	116(18)	273(17)
Phone call	285(29)	203(31)	488(30)
Home visit	528(55)	328(51)	856(53)

^1^ 1 Kwacha = 0.1 USD

### Linkage to care

Respondents were asked to imagine that they had taken an HIV self-test and their result was positive. Out of the 1,617 respondents, 85% (95%CI 83–86) said they would be willing to link to care within a week of having a positive result (Table 2). In univariable analyses, both high income earners (K2,000+) (Unadjusted OR = 0.61; 95%CI: [0.41, 0.89]; p = 0.011) and those who are somewhat or not comfortable with HIV selftesting (Unadjusted OR = 0.49; 95%CI: [0.37, 0.65]; p<0.001) were associated with lower odds of intention to link to care (Table 3). However, there was no evidence of association between those with no prior HIV test and reduced odds of intention to link to care (Unadjusted OR = 0.77; 95%CI: [0.54, 1.11]; p = 0.164) (Table 3). In a multivariable logistic regression model, income >2,000 Kwacha (USD 200) per month versus income < 2,000 Kwacha (Adjusted odds ratio (AOR) = 0.59; 95%CI: 0.40 to 0.88; p = 0.009) and never versus prior HIV testers (AOR = 0.54; 95%CI: 0.32 to 0.91; p = 0.020) were independently associated with reduced odds of intention to link to care (Table 3). The independent effect of acceptability had diminished when prior HIV testing was included in the final model, whereas the independent effect of prior testing became significant. Employment status was collinear with income and so was dropped from the multivariable logistic regression model. To explore the apparent diminished effect of acceptability and prominence of prior HIV test, we manually modelled the combinations of income, prior HIV testing and acceptability. We observed that having income and acceptability in the multivariable model diminished the effect of acceptability while the effect of income remained the same. But having income and prior HIV testing in the multivariable model increased the effect of prior HIV testing while the effect of income (again) remained the same. Having acceptability and HIV testing in the model did not change the effect of either of them. Thus, prior HIV testing appears to have a suppressor effect on the relationship between acceptability and intent to link to care [15, 16].

**Table 2:** Percentage of respondent aged 16–49 years who reported willing to link to care if HIV self-test result was positive and follow up strategy.

Characteristics	Number (% of total)	Intent to link to care within a week if self-testing result is positive:			Preferred follow up home visit strategy		
		Number (%)	95%CI	Chi2 p-value	Number (%)	95%CI	Chi2 p-value
**Age (Years)**							
16–24	571(35)	487(85)	[82, 88]	0.80	307(54)	[50, 58]	0.13
25–34	626(39)	532(85)	[82, 88]		313(50)	[46, 54]	
35–49	420(26)	352(84)	[80, 87]		236(56)	[51, 61]	
**Education N (%)**							** **
No Education	47(3)	41(87)	[74, 94]	0.34	31(66)	[51, 78]	**0.01**
Primary Education	284(18)	231(81)	[76, 85]		171(60)	[54, 66]	
Secondary Education	1056(65)	904(86)	[83, 88]		538(51)	[48, 54]	
Tertiary Education	229(14)	194(85)	[79, 89]		115(50)	[44, 57]	
**Monthly income (Kwacha)**^**1**^							
<500	175(22)	161(92)	[87, 95]	**<0.01**	101(58)	[50, 65]	0.24
501–1000	227(28)	186(82)	[76, 86]		111(49)	[42, 55]	
1001–2000	170(21)	142(83)	[77, 88]		91(53)	[46, 61]	
2001–5000	181(22)	138(76)	[69, 82]		87(48)	[41, 55]	
>5000	52(6)	44(85)	[72, 92]		23(44)	[31, 58]	
**Employment status**							
Employed	371(23)	316(85)	[81, 88]	0.10	183(49)	[44, 54]	0.27
Self-employed	463(29)	379(82)	[78, 85]		253(55)	[50, 59]	
Unemployed	783(48)	676(86)	[84, 89]		420(54)	[50, 57]	
**Prior HIV Testd**							
Yes	1375(85)	1173(85)	[83, 87]	0.16	729(53)	[50, 56]	0.88
No	242(15)	198(82)	[76, 86]		127(52)	[46, 59]	
**HIVST Acceptability**				** **			** **
Very Comfortable	1198(75)	1048(87)	[85, 89]	**<0.001**	632(53)	[50, 56]	**0.07**
Somewhat Comfortable	263(16)	204(78)	[72, 82]		129(49)	[43, 55]	
Not Comfortable	144(9)	111(77)	[69, 83]		88(61)	[53, 69]	
**Total**	**1617**	**1371(85)**	**[83, 86]**	** **	**856(53)**	**[50, 55]**	** **

^1^ 1 Kwacha = 0.1 USD

**Table 3:** Factors independently associated with odds of intention to link to care among respondent aged 16–49 years, Lusaka, Zambia.

	Crude Odds Ratio [95%CI]	P-value	Adjusted Odds Ratio [95%CI]	Adjusted p-value
**Monthly income (Kwacha)**^**1**^				
<2,000	reference	**0.011**	reference	**0.009**
2,000+	0.61[0.41, 0.89]		0.59[0.40, 0.88]	
**Employment status**				
Employed	reference	0.107		
Self-employed	0.79[0.54, 1.14]			
Unemployed	1.10[0.77, 1.56]			
**Prior HIV Test**				
Yes	reference	0.164	reference	**0.020**
No	0.77[0.54, 1.11]		0.54[0.32, 0.91]	
**HIVST Acceptability**		** **		** **
Very Comfortable	reference	**<0.001**	reference	**0.149**
Somewhat/Not Comfortable	0.49[0.37, 0.65]		0.74[0.49, 1.12]	

^1^ 1 Kwacha = 0.1 USD

FGD participants believed that going to the clinic after getting a positive result from a self-test was the right thing to do. They understood that one needed to seek advice and medication from the clinic if they tested positive:

“When you test yourself and find that you are sick, you just go to the clinic and you tell them you are sick. They will not just let you go without talking to you … because those people know how to comfort us. They will sit you down and tell you things that will comfort you and that you are not the only one nor are you the last one.”

–Female FGD Rural.

Some group members in each group felt that social support from the clinic or from friends was needed for a person to deal with an HIV positive result from a self-test. Some participants noted that people react differently when they receive a positive result voicing fears that people may choose to start infecting others intentionally, commit suicide and endure partner violence as below:

“I recommend people to use it (self-test) but I would prefer if there was pre and post counselling, because I understand knowing your status if you are positive comes with a burden for lack of a better term. I think that pre and post counselling is very important before you know your status because sometimes if you are alone you can commit suicide [as a] consequence of knowing your status. When you are alone you need people around or maybe perhaps you can take it with your partner so that they give you the support when you find out that your status is positive and encourage you to go to the clinic.” Male FGD Peri-urban

On the other hand, some group members felt that pre-test counseling from the clinic or some form of post-test advice from a trusted friend was required in order for a person to link to care. Some participants noted that people react differently when they receive a positive result voicing fears that people may choose to start infecting others intentionally, commit suicide and endure partner violence as below:

“If a person is positive they need to find people who can help [them], so that they can be comforted and not have the feeling of saying ‘why have I been found positive or what can I do?’… Others commit suicide and they tell themselves they are better off dying than suffering with the illness. So they should be counseled so that they can understand.”–Male FGD Rural

“Us as people we have two kinds of attitudes. The first one you will find that I discover I am sick and I need to go to the clinic. Secondly others … will start misbehaving like a mad [person] that infects anyone …[and] makes everyone sick.”–Male FGD Urban

“The major thing that I have noticed is the fact that people are different. You can test yourself at home and find that you are sick and not do anything about it. Then you start misbehaving because you are sick and you start telling yourself that you [will] infect others”–Female FGD Rural

### Follow up strategy

Overall 53% (95%CI = 50 to 55) (Table 2) of respondents reported preferring a home visit from a health care worker, and 30% preferred a phone call, followed by 13% who preferred SMS (Table 1). Preference for a home visit appears to vary between the levels of education with 66% of those with no education preferring a follow-up home visit compared to 50% of those with tertiary education (P-value = 0.01) (Table 2).

Participants in FGDs reported more positive sentiments for home visits compared to phone call and text message. More than once, they expressed that home visits were a more personal approach that would allow for effective communication. Participants said a home setting was more comfortable than a health facility setting that allows people to express themselves freely. Some participants preferred to be visited because they felt that people who know them can hear them disclose their status at the clinic because it was not private. Further, it was reported that testers can give an excuse on the phone about linking to care, but would feel embarrassed to give an excuse when they are visited at home:

“I think both modes (phone and home visit) of talking to your counsellor are important, but if you want an effective communication, its better people come to your home so that you build trust and strong relationship—Male FGD Peri-urban

Other participants expressed concern about home visits. Female participants reported that the community may perceive being visited by a male health care worker would appear as though they were being visited by a boyfriend which may bring problems in their marriages. Others commented that being visited by a health care worker was an indication that a person is HIV positive:

“… Other people will start having questionable thoughts, like who is that person visiting her in her house … If it is a man who has visited her and the husband comes [home], he might think that the visitor is her boyfriend and they would start fighting.”—Female FGD peri-urban

## Discussion

In the first study of HIVST in Zambia, 85% of those participating in a representative survey reported that they would intend to link to care within a week if they used an HIVST and found they were positive. Linkage to care within a week is associated with income status and whether one has ever tested. Additionally, 53% preferred home visits as the most suitable follow up strategy for linkage to care than phone call (30%) and SMS (17%). Home visits by a health care worker were preferred because patients could form trust and communicate effectively, although the sensitivity to the gender of the health care worker was an important consideration for some.

Linkage to HIV care can be considered as accessing a health care provider through a clinic at four different stages, including i) enrolment into care after diagnosis, ii) to determine antiretroviral therapy eligibility, iii) to initiate ART and iv) adherence to ART [17]. Facility based HIV testing provides an opportunity for HIV positive patients to receive post-test counselling and immediately enrol into HIV care through ART centres within the clinic. In this study participants understood linkage to care as counselling and enrolment into care after diagnosis of HIV and were fully aware that the onus was on them to be linked to care after using a self-test. With this understanding 85% potential HIVST users had the intention to link to care within a week of knowing their status. Linking to care within a week can be considered optimal behaviour given that linkage to care is defined as “having visited a health care provider within 30days of being diagnosed with HIV” [18]. In order to encourage linkage to care, half of the respondents preferred to be followed up through home visits and one third preferred phone calls. SMS was the least preferred because it was difficult to establish a relationship using this platform. Respondents felt it was important for them to be able to express their concerns and feelings after receiving a positive result, which would not be possible through SMS.

The multivariable logistic regression showed that those who earned a monthly income of less than 2,000 Kwacha (USD 200) and those that have previously tested for HIV were more likely to intend to link to care. The finding that the lowest income individuals were more likely to intend to seek care is important given that many of the HIV prevention and treatment services in Zambia are targeted towards public health facilities accessed largely by those in lower income brackets and where the burden of HIV lies [19]. Our finding may reflect less direct experience with HIV and the need for linkage to care among some with higher incomes and a potential need to address this in future implementation work. Furthermore, the finding of higher linkage intention among those with a prior HIV test could be a consequence of the relationship between positive health seeking behaviours and having repeated tests overtime [20]. Based on their testing history, HIV testers may feel more confident to link to care if found positive. These findings are consistent with the findings from a cross sectional survey conducted in the United States where participants who had not received prior testing were more likely to delay in linking to care [21]. While testing history is important it’s not relevant for linkage to care strategies as they only target people who have ever tested HIV positive. However, HIV testing strategies should seek those that have never tested as they may be a harder population to convince on the benefits of knowing ones status.

There is limited data showing that the poor earning less than 2,000ZMR in peri-urban areas are more likely to link to care than those earning more. Further studies should be conducted to understand this link. A systematic review conducted to understand the barriers associated with linkage to care found that transport costs to the clinic was an economic factor that hindered linkage to care [22]. Therefore, while the poor may intend to link to care they may not afford costs associated with accessing care from the clinic. With respect to the association between prior HIV testing and linkage to care, its important to note that they are many other factors that influence linkage to care. These are not only at individual level but at facility, community and structural levels [23]. Additionally, evidence suggests that suffering illness is a strong determinant of linkage to care [21, 23]. Hence, people that know they are HIV infected but feel generally healthy are more likely not to link to care. For these, exploring follow-up strategies using phone calls and home visits can enhance linkage to care. Both methods have been used before and proven effective in improving patent enrolments and retention in HIV care [24, 25] and should be adopted in following up self-test users. Care needs to be placed on the presentation of community health workers as they make home visits so as to avoid suspicion.

There are limitations to this study. Firstly, linkage to care is expressed as an intention and is not measured as an actual behaviour. This is because the study was a feasibility study conducted prior to the introduction of the HIV self-test in Zambia. Intention to link to care may not translate into actual linkage to care behaviour. Thus there is need to measure linkage to care when the self-test is rolled out in Zambia. Furthermore, the study population is representative of Lusaka Province only and not of the entire country. Lastly, we did not conduct in-depth interviews with those not willing to link to care within a week. Future studies should examine this group, to gain insights into the barriers towards linkage to care amongst potential HIV self-test users.

HIV policy guidelines need to be revisited to include strategies that enhance linkage to care for those who test positive from the self-test but do not link to care. These strategies should specifically include those that are in a higher income brackets, who may not be reached through public health facilities or community sensitization events. Designing follow-up strategies is complex with the HIVST because the onus lies on potential HIVST users to report their result at the health facility. Relying solely on the user to report their result may not be effective given the current low linkage to care figures. Thus, it is necessary to carefully design distribution points for the pick-up of self-tests that will allow user information to be retained for the purpose of linkage to care strategies.

## Conclusion

In this representative cross sectional survey of Lusaka Province, intention to link to care within a week after a positive result from an HIVST was reported by almost nine out of ten of the population. Prior testing for HIV and low-income status had increased odds of intention to link to care. Home visits and phone calls are preferred follow-up methods for those that would not link to care after receiving a positive result from an HIVST. These findings are important for the development of HIVST policy guidelines and implementation strategies for linkage to care.

## Supporting information

S1 FGD Guide (DOCX)
