# Supplementary material for: Understanding linkage to care with HIV self-test approach in Lusaka, Zambia - A mixed method approach
Source: PLoS One. 2017 Nov 17;12(11):e0187998. doi: 10.1371/journal.pone.0187998 (PMC5693414; doi:10.1371/journal.pone.0187998)
Supplement: S1 FGD Guide — (DOCX) [file pone.0187998.s001.docx]

**Introduction Statement**

Hello Everyone! It’s very nice to have all of you here; we will have a nice discussion today. My name is _____________________ and I’ll be leading the discussion today. My friend here will introduce himself/herself.

We are having this discussion to fine out your views on a new method of knowing your HIV results called HIV Self-Testing. This test has not yet been introduced in Zambia. The Government of Zambia would like to know your views before introducing it in Zambia. The discussion will take a maximum of 1.5hours.

Now we would like to get to also know you. I would like to remind you that anything we discuss here will be kept confidential. Due to this we will not be getting your real names. Please pick a name in the box and introduce yourself using the name you pick. Tell us something about what you like to do when you have some free time.

Than you now let’s start.

| **Discussion Topic** | **Questions Guide** |
| --- | --- |
| **Knowledge and behaviour on HIV testing** | 1. Can anyone of you tell me what you know about HIV testing? 2. In your view, what are the advantages and disadvantages of HIV testing? 3. We all know people who have had an HIV test and people who have NOT taken an HIV test. What do you think are the main factors that stop some people from taking a test? [The facilitator should emphasise that none has to reveal their own testing choices unless they want to share with the group; and that the group should keep all information confidential irrespective of whether people provide personal experience |
| **HIV Self Testing** | *Show the group a video of what an HIV self-test. Ask the following questions:*   1. What do you understand by the video I’ve just shown you? Allow for all to respond. 2. Has anyone here ever heard about HIV self-testing before today? Explain further if group is still not clear and give these specific details:    1. It's a saliva test shows you whether you are positive or negative. The HIV virus is not present in the saliva, the tests detect the antibodies that fight the HIV virus.    2. It can be done in the comfort of your own home or anywhere else you feel is comfortable.    3. It can be done in private    4. It takes 20mins before you can read the result 3. Now that I have explained to you further, tell me what your thoughts are on HIV self-testing. 4. What are your thoughts on others being present while the test is being taken? 5. Based on what you’ve seen here, do you feel that that you can correctly use the HIV self-test yourself? Why or why not? Do you think other people in your communities can correctly use the self-tests? 6. Which types of people do you feel would like to use the self-test between:    1. Single and married people    2. Men and women   Who else do you think would be happy to use the test? Why do you say so?   1. Are there any fears/concerns that come to your mind concerning the use of self-testing? 2. Do you think self-testing should be promoted in Zambia? Why or why not? |
| **Confirmatory testing and linkage to care** | After one has taken a self-test at home, regardless of whether the result is positive + or negative – he/she is required to go to the nearest clinic to see a health care provider to confirm the test. The health care provider will re-do the test and if it is positive will start the client on HIV drugs. .  Show the participants the following storyboards beginning with Scenario A and B.  Start by passing round scenario A to everyone in the group. Then ask a maximum of two people what they think the story is telling. End by summarising what the scenes are showing.  ***Scenario A* -** she had no counseling before taking the test, she tested at home she received no personal follow-up, she reads a brochure explaining that a positive test needs to be repeated at the facility.  ***Scenario B*** – she had counseling before taking the test, she tested at home, she received no personal follow-up, and she receives an SMS explaining that a positive test needs to be repeated.  **Now ask:**   1. What is the difference between the person that received counseling and the person that did not receive counseling? 2. What is the difference in the way they will react if they find out that their result is negative? 3. What is the difference in the way they will react if they find out that their result is positive? 4. Which method will better convince this person to go to the clinic to confirm their result between the brochure and the SMS?   Now pass round scenario C and D. Ask a maximum of two people what each scenario is trying to say? Summarize by giving the right details.  ***Scenario C*** – she had counseling before taking the test, she tested at home, and she receives a call from a CHW explaining that a positive test needs to be repeated.  ***Scenario D*** – she had counseling before taking the test, she testes at home and she receives a visit from a CHW reminding her that a positive test needs to be repeated.  **Now ask:**   1. What difference will there be in reaction between the person that receives the call and the person that receives the visit? 2. How do you think different people would react to a positive result after taking the self-test at home? |
| **Demonstrating on how to use the test** | 1. In your own view, what do you think is the best way to teach individuals and/or couples about HIV self-testing?   *Now you will distribute the paper instructions to three groups and the video to the other three groups. Ask them to spend 2mins looking at them. Now ask:*   1. For each step shown on the paper-based/ video instructions ask:    - What is it trying to communicate?    - Is it easy to understand?    - Is this doable?    - Is it difficult?  - Think about people in your community. If they received the instruction sheet alone, what would you say about their levels of understanding and their ability to perform the test correctly?  1. *Probe for:*    1. Who will mostly likely understand these instructions?    2. Who would you feel comfortable with and trust to communicate this information?    3. Is there anything else you would add or take out from these instructions? |
| **Access** | 1. If you wanted to access a self-test, where do you feel comfortable going to get it? Why? 2. Do you think the church can be a distribution point for the test? 3. What about workplaces? 4. What are your thoughts on local drug stores supplying the HIV self test for free? Why do you say so? |
